# A Method for Preparing Morphologically Preserved Wildlife Fecal Specimens for Long‐Term Ecological Studies

**DOI:** 10.1002/ece3.72931

**Published:** 2026-01-11

**Authors:** Jiahao Zhang, Dongling Zhang, Xinrui Xu, Yunqiao Zhang, Qiang Dai

**Affiliations:** ^1^ Chengdu Institute of Biology, Chinese Academy of Sciences Chengdu China; ^2^ University of Chinese Academy of Sciences Beijing China; ^3^ Yingjing Conservation and Management Station of Giant Panda National Park Ya'an Sichuan China; ^4^ Crop Research Institute of Sichuan Academy of Agricultural Sciences/Crop Germplasm Innovation and Genetic Improvement Key Laboratory of Sichuan Province Chengdu China

**Keywords:** fecal specimen preparation, long‐term storage, noninvasive sampling, wildlife monitoring

## Abstract

Wildlife feces are a valuable noninvasive resource in ecological and conservation research. However, traditional preservation methods are unable to maintain morphological integrity while simultaneously preserving the biological and chemical composition of fecal samples. This study introduces a novel method for the preparation of fecal specimens through a multistep infiltrated process using sodium carboxymethyl cellulose, sodium benzoate, clotrimazole, ethanol, pyrethroid emulsion, and polyvinylpyrrolidone solution. The entire procedure takes approximately 7 days to complete one batch of specimens. The specimens produced using this method exhibited high mechanical strength, ensuring durability and resistance to handling damage. Over an 18‐month storage period, the preserved specimens retained their external morphology and showed no signs of mold or insect damage. DNA integrity was well maintained, with a 100% success rate in DNA extraction, and species identification based on preserved specimens was identical to that obtained from the corresponding pre‐preservation feces. Furthermore, heavy metals such as chromium, arsenic, and lead were successfully detected in fecal samples from different species. By allowing long‐term preservation of fecal samples, this method converts feces from a transient diagnostic tool into a durable resource for monitoring biodiversity. It can broaden the spatial and temporal applicability of fecal samples and strengthen their role in ecological research and biodiversity conservation.

## Graphical abstract

This study introduces a novel multistep immersion method to preserve wildlife fecal samples, maintaining morphological integrity, DNA stability (100% extraction success, > 99% BLAST match rates), and resistance to mold/insect damage for 6 months. The technique also enables heavy metal detection, transforming feces into a durable resource for long‐term ecological monitoring and biodiversity conservation across spatiotemporal scales.

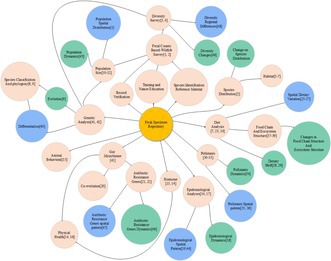

## 1 Introduction

As noninvasive biological samples, wildlife feces have played a significant role in ecological and conservation biology research (Mondol et al. 2009; Węgrzyn et al. 2018; Navarro‐Castilla et al. 2023). Since Seton (1925) introduced “scatology” to mammalogy, fecal research has expanded across various scientific disciplines (Hartman et al. 1958). Fecal samples are particularly valuable in field surveys where direct observation or population estimation is challenging, especially for elusive or rare species (Palomares et al. 2002). Modern scatological studies address a broad spectrum of ecological questions, including biodiversity (Boyer et al. 2015), habitat use (Bearer et al. 2008), and population surveys (Monterroso et al. 2019). Additionally, feces also provide valuable information for studies on behavior (Navarro‐Castilla et al. 2019), genetics (Oja et al. 2017), and physiology (Barja et al. 2007).

Despite their wide range of applications, two challenges constrain the reliability and reusability of fecal data. First, accurate species identification remains a persistent difficulty, especially in regions with high biodiversity, where feces from different species often exhibit similar morphology (Hawlitschek et al. 2018), while those from the same species may vary substantially due to dietary differences (Magondu et al. 2023). Such misidentifications can lead to alarmingly erroneous outcomes, raising concerns about the reliability of feces‐based field surveys (Costa et al. 2017; Spitzer et al. 2019). Second, existing preservation protocols focus primarily on DNA extraction, including frozen storage (Shores et al. 2015), ethanol preservation (Murphy et al. 2002), and silica desiccation (Murphy et al. 2000), while largely overlooking the need to maintain morphological and chemical integrity. Consequently, fecal samples rarely function as long‐term verifiable specimens suitable for retrospective or comparative analyses.

We believe that fecal specimens that preserve both morphological features and biological as well as chemical composition represent a valuable resource for ecological research and biodiversity conservation. Specimens with genetically confirmed species identity can serve as reliable training references, helping to reduce misclassification in the field while also providing verifiable records that support data validation, repeatability, and reproducibility. Moreover, preserved fecal specimens hold great promise as long‐term archives of ecological processes, offering insights into environmental degradation (Malaney and Cook 2018), pollutant exposure (Tran et al. 2015), dietary shifts (Blumenthal et al. 2012), pathogen transmission (Delahoy et al. 2018), and historical ecosystem change (Wandeler et al. 2007; Burrell et al. 2015), as well as other emerging applications as research methods evolve. This role parallels that of museum specimens, which have become indispensable for reconstructing past ecological and environmental conditions (Wandeler et al. 2007; Staats et al. 2013; Burrell et al. 2015). Additionally, fully sterilized fecal specimens can serve as valuable materials for nature education, providing an engaging, hands‐on approach to promote experiential learning.

This study presents an innovative approach for preparing mammalian fecal specimens that preserves both morphological features and biological as well as chemical composition, thereby providing a reliable foundation for verification and future analyses. To evaluate the effectiveness of this method, we applied it to fecal samples of various carnivores and herbivores, evaluating their morphology, heavy metal content, and DNA Quality after extended storage. This approach not only ensures the long‐term preservation of fecal specimens but also substantially expands their utility in ecological and conservation research, particularly under the accelerating pressures of global environmental change.

## 2 Materials and Methods

### 2.1 Field Collecting

Fecal samples were collected from forested habitats in the Yingjing region of the Giant Panda National Park, China, covering six representative wildlife species— *Elaphodus cephalophus* (herbivore), *Ailurus styani* and *Macaca thibetana* (omnivores), and *Hystrix brachyura* , *Sus scrofa* and *Prionailurus bengalensis* (carnivores)—to encompass a broad range of dietary habits and fecal characteristics. During field collection, only intact feces were sampled, and their original morphology was photographed for documentation before being placed in sampling containers. Fresh feces, generally characterized by intact morphology, a moist or glossy surface, and no signs of desiccation, cracking, or fungal growth, were prioritized for DNA extraction.

Each container was prepared with a layer of drying silica gel at the bottom, followed by the fecal sample, which was positioned to maintain its natural shape, and then completely covered with additional silica gel. Approximately 100 g of silica gel was used per 5 g of fecal material, with the amount adjusted upward for samples with higher moisture content. To prevent mechanical damage during transport, a layer of acrylic fiber or sterile cotton was placed above the silica gel as cushioning. The containers were then sealed with airtight lids and wrapped in cling film to prevent content dispersal and protect against external moisture and contaminants. All samples were transported to the laboratory for further preparation within 1 month of field collection. During transport and temporary storage, the condition of the silica gel was inspected weekly and replaced when it became ineffective.

### 2.2 Preparing Procedure

First, fecal samples were saturated with a 4% sodium carboxymethylcellulose solution and air‐dried for 8 h at room temperature (approximately 24°C). Care was taken to avoid high drying temperatures, which could cause cracking and compromise morphological integrity. Depending on the sample size, the saturation and drying steps may need to be repeated three or more times to ensure thorough fixation. Next, the fecal specimens were immersed sequentially in ethanol solutions of 75%, 85%, 95%, and 100% concentration, with each step followed by air drying for 2–4 h. Subsequently, antibacterial and antifungal treatments were performed using 0.1% sodium benzoate in ethanol and 0.5% clotrimazole in acetone. Each treatment was applied at least three times, followed by air drying for 8 h after each step. An additional overnight air drying was conducted to ensure complete evaporation of acetone.

**FIGURE 1:**
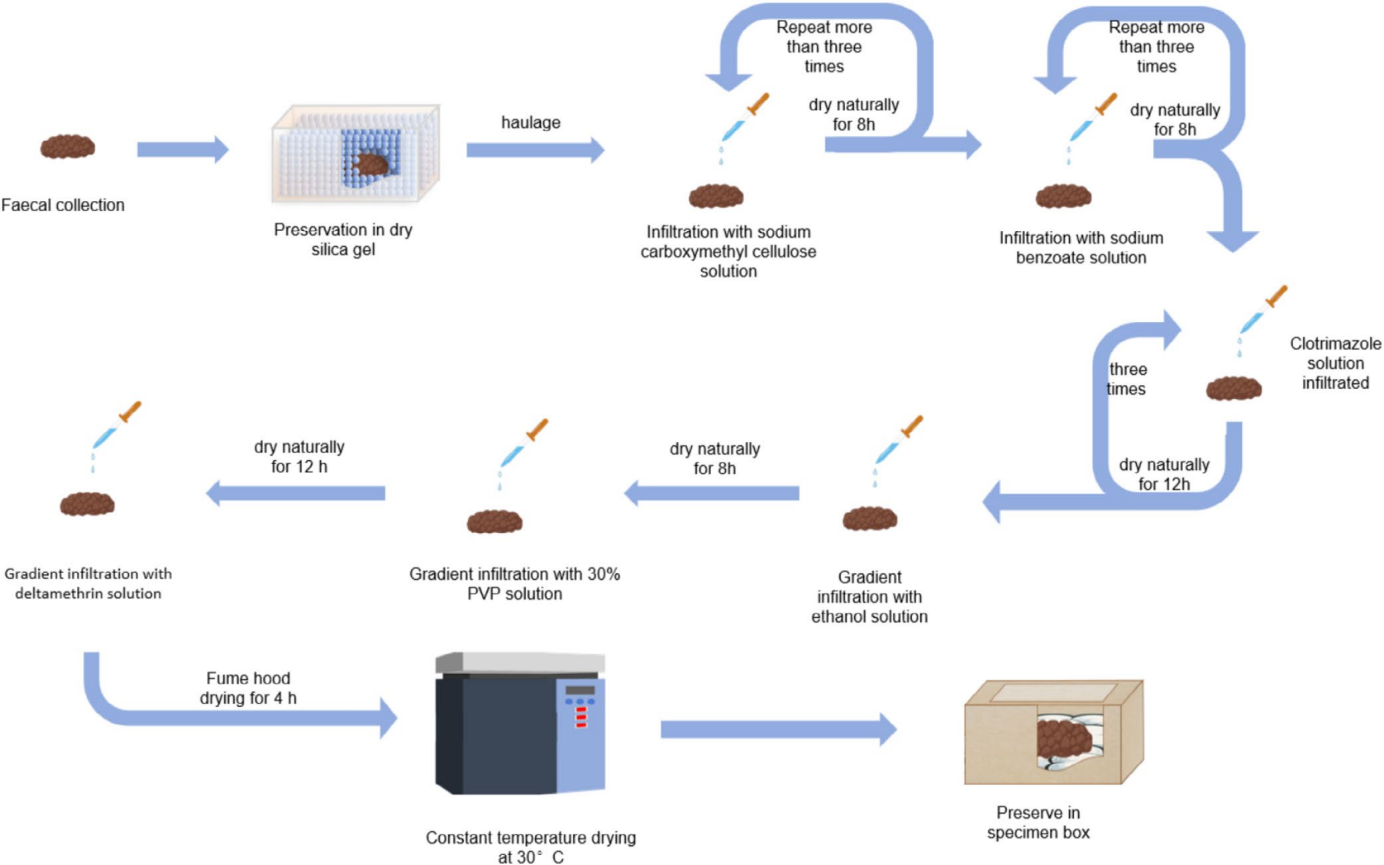
Workflow for fecal sample preparation.

To reinforce structural stability, the specimens were then infiltrated with a 30% polyvinylpyrrolidone (PVP) solution and air‐dried for 1–2 days. Afterward, they were infiltrated with a 2.5% pyrethroid emulsion for 3 min to prevent insect infestation and dried in a fume hood for 4 h. Finally, the fecal specimens were oven‐dried at 30°C to remove residual moisture and ensure complete stabilization (Figure 1).

The entire six‐step procedure was completed within approximately 7 days. Because the method involves sequential immersion steps, multiple specimens could be processed simultaneously.

### 2.3 Specimen Preservation

To prevent insect infestation, one to two mothballs were first placed at the bottom of the sample box, followed by a layer of acrylic fiber and color‐changing silica gel. The fecal specimens were then placed on top (Figure 1). Finally, the specimens were labeled, recorded, and archived in accordance with museum protocols.

### 2.4 Mechanical Properties

Shore hardness was measured using an LX‐A dual‐needle Shore durometer (GOYOJO Industrial Technology, Shenzhen, China) under static compression conditions, with five replicate measurements taken for each specimen. This test assesses a material's resistance to indentation and is widely used to assess the mechanical properties of polymers, elastomers, and biological specimens (Falanga and Bucalo 1993; Basfar 1997). The LX‐A durometer, designed for soft materials, applies a standardized force to determine the firmness and structural integrity of fecal specimens. Hardness values range from 0 (extremely soft, gel‐like materials) to 100 (hard rubber‐like materials), providing a quantitative measure of specimen durability and confirming their suitability for long‐term storage and handling.

### 2.5 DNA Quality Assessment

A 220–260 mg sample was carefully scraped from the surface of each feces using a sterile scalpel and transferred to a 2 mL centrifuge tube for further processing. To avoid cross‐contamination, forceps, scalpel, Petri dishes, and bench surfaces were immediately sanitized with ethanol or replaced after handling each sample. DNA was extracted with the QIAamp Fast DNA Stool Mini Kit (Qiagen) according to the manufacturer's instructions. The total DNA extracted was stored at −20°C until further use.

Amplification of a partial sequence of the mitochondrial 16S ribosomal RNA (16S rRNA) gene was performed using the forward primer F (5′‐GAGAAGACCCTA TGGAGC‐3′) and reverse primer R (5′‐ATAGAAACCGACCTGGAT‐3′) for Sanger sequencing. This marker set proved effective for species‐level identification across mammals with diverse dietary guilds (Xiong et al. 2016; Wang et al. 2025). PCR reaction mixture contained 2 μL DNA template, 10 μL Taq PCR Master Mix (Shenggong Biological Engineering Co. Ltd.), 0.8 μL each forward and reverse primer (10 μM), and 6.4 μL nuclease‐free water (ddH_2_O), for a total volume of 20 μL. Specific PCR protocols were used for each gene locus. For 16S rRNA, the conditions were: initial denaturation at 95°C for 2.5 min, followed by 35 cycles of 95°C for 30 s, 50°C for 30 s, and 72°C for 1 min, with a final extension at 72°C for 10 min.

Species identification was based on a sequence similarity threshold of ≥ 97% (Hebert et al. 2003). The best‐matched sequence for each sample, cross‐validated against known species distributions in the sampling region, was considered as the corresponding species identification. DNA was first extracted from fecal samples for species identification prior to the preservation procedure. After 6 and 18 months of storage, DNA was re‐extracted from the same samples to evaluate the effects of preservation duration on DNA integrity and identification accuracy.

### 2.6 Heavy‐Metal Detection

Heavy‐metal concentrations were measured in paired fecal samples collected before and after 1 month of specimen preservation. To determine the concentrations of chromium (Cr), copper (Cu), zinc (Zn), arsenic (As), cadmium (Cd), lead (Pb), and mercury (Hg), the fecal samples were dried at 50°C, ground, and passed through a 0.15 mm metal sieve. A total of 300 mg of fecal samples was digested in a 3:1 (v/v) mixture of HNO_3_ and HClO_4_ (Jin et al. 2023). After digestion, the solutions were filtered and diluted to a final volume of 50 mL with ultrapure water. Elemental concentrations were quantified using inductively coupled plasma mass spectrometry (ICP‐MS).

## 3 Results

### 3.1 Morphology and Hardness

**FIGURE 2:**
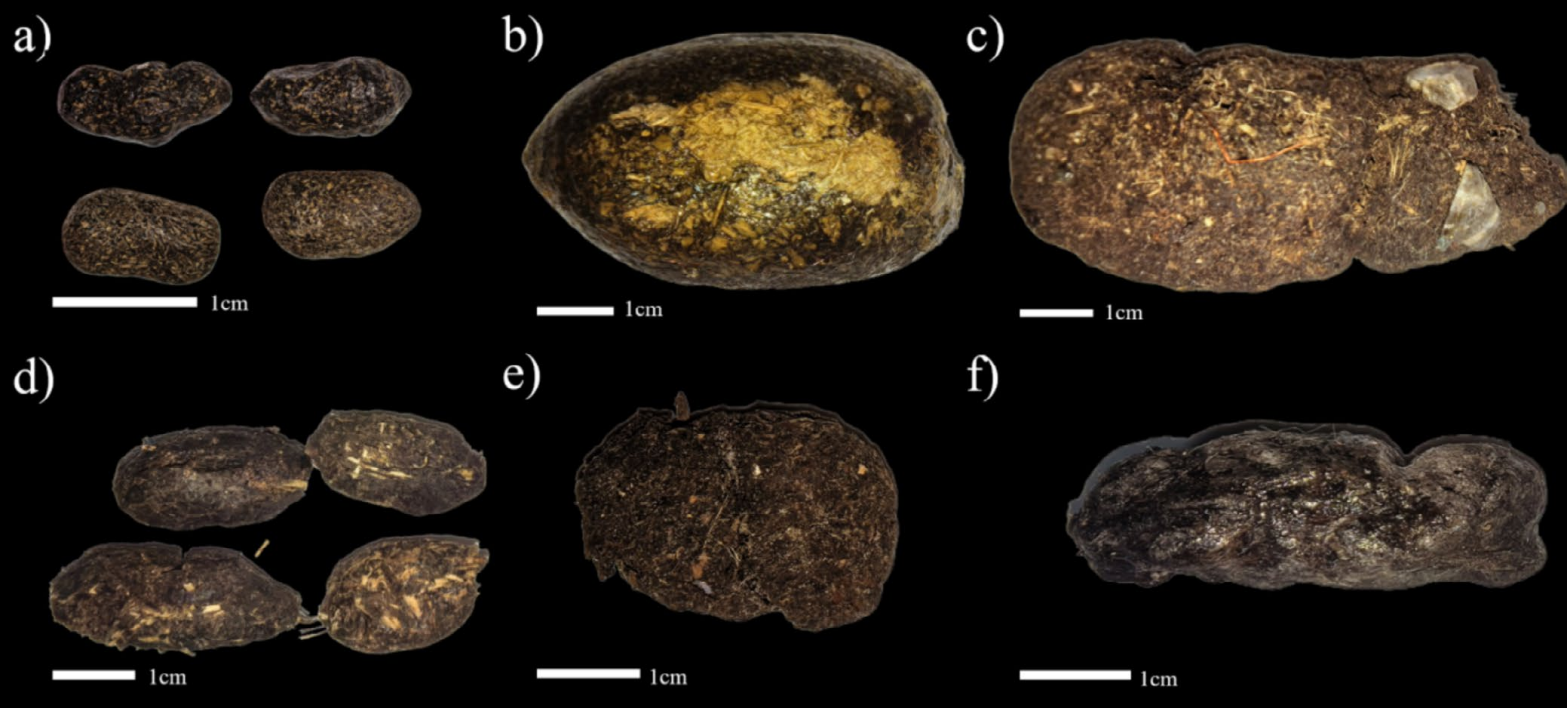
Photos of fecal specimens of (a) *Elaphodus cephalophus* , (b) *Ailurus styani*, (c) *Macaca thibetana* , (d) *Hystrix brachyura* , (e) *Sus scrofa* , and (f) *Prionailurus bengalensis* .

Overall, the preserved specimens exhibited excellent structural integrity and resistance to physical damage, effectively maintaining the original morphology of the feces. As shown in Figure 2, the specimens retained the natural color and surface sheen of pre‐preservation feces samples, with clearly visible animal hairs or plant fibers, indicating that key external characteristics were well preserved. Over the course of 18 months, no evidence of decay, mold growth, or insect infestation was detected, further confirming the stability and effectiveness of the preservation method.

Shore hardness tests showed that the preserved specimens were generally substantial, with most exhibiting hardness values between 79 and 85 (Table 1), comparable to firm rubber or dense leather, demonstrating strong structural integrity. An exception was the *A. styani* samples, whose specimens had a notably lower mean hardness of 36.02, similar to that of soft rubber or an eraser, suggesting a much softer and less rigid structure. This reduced hardness may be attributed to the species' diet, which consists largely of bamboo leaves, resulting in feces with a naturally looser and less compact structure.

**TABLE 1:** Shore hardness measurements of fecal specimens.

Fecal specimens	Hardness (Shore A)		
	Mean	Range	Standard deviation
*Elaphodus cephalophus*	84.92	81.4–89.9	3.03
*Ailurus styani*	36.02	25.6–47.1	9.08
*Macaca thibetana*	79.36	68.8–84.5	5.63
*Hystrix brachyura*	82.46	72.8–90.0	6.51
*Sus scrofa*	85.04	75.5–93.0	6.13
*Prionailurus bengalensis*	81.38	74.3–89.4	5.67

### 3.2 DNA Quality Assessment

**FIGURE 3:**
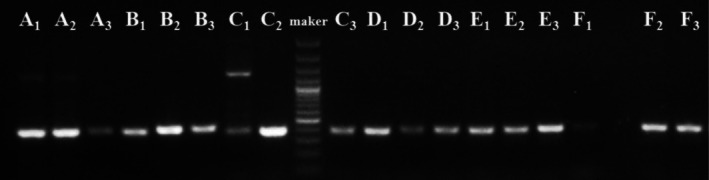
Gel electrophoresis results of DNA extracted from fecal samples. Species are indicated by letters: (A) *Elaphodus cephalophus* , (B) *Ailurus styani*, (C) *Prionailurus bengalensis* , (D) *Macaca thibetana* , (E) *Hystrix brachyura* , (F) *Sus scrofa* . Time of DNA extraction is indicated by numeric suffixes: (1) pre‐preservation feces, (2) after 6 months of preservation, (3) after 18 months.

A total of six fecal samples were analyzed to assess DNA quality, including DNA extracted from pre‐preservation feces samples and from specimens preserved for 6 and 18 months. Gel electrophoresis showed that DNA was successfully extracted from all preserved fecal samples (Figure 3). A faint band was observed in Sample F_1_, indicating a relatively low DNA concentration. Nevertheless, this did not hinder subsequent PCR amplification or sequencing, as the resulting reads met the quality thresholds required for reliable taxonomic identification.

Sequence homology was evaluated using BLAST against the NCBI database. Table S1 summarizes the sequence data and key metrics for fecal samples preserved for 0, 6, and 18 months, including total score, expect value, query cover, and percent identity. The consistently low expected values indicate high sequence reliability. All samples exhibited ≥ 97% identity, except for the *P. bengalensis* sample at 6 months, which showed a lower identity (88%) but returned to 97.87% at 18 months. The temporary drop at 6 months was most likely due to subsampling from an unsuitable part of the feces, which reduced DNA quality, rather than to degradation during preservation. These results confirm that DNA extracted from preserved specimens remains highly consistent with that from pre‐preservation fecal samples, supporting reliable species identification.

### 3.3 Heavy Metal

ICP‐MS analysis detected all six targeted metals (Cr, Cu, Zn, As, Cd, Pb) in pre‐ and post‐preservation samples, confirming the suitability of preserved feces for elemental quantification (Table 2). Although the concentrations differed across species, paired comparisons revealed only minor and non‐directional variation following preservation. For example, in *E. cephalophus* , As levels decreased from 0.87 to 0.64 mg/kg, while Cd increased from 0.23 to 0.29 mg/kg. In *H. brachyura* , Zn declined from 163.41 to 105.39 mg/kg, whereas Cd rose slightly from 0.25 to 0.31 mg/kg. These results suggest that the preservation process does not systematically alter the levels of heavy metals.

**TABLE 2:** Heavy metal content in fecal specimens.

Fecal source	Timepoint	Heavy metal elements (mg/kg)					
		As	Cu	Zn	Cr	Cd	Pb
*Elaphodus cephalophus*	Before	0.87	20.64	202.74	4.89	0.23	2.24
	After	0.64	16.61	158.66	4.37	0.29	2.67
*Budorcas taxicolor*	Before	0.34	12.48	147.96	2.75	0.18	1.16
	After	0.25	9.31	109.81	2.22	0.16	1.55
*Macaca thibetana*	Before	0.22	31.59	58.76	1.41	0.02	1.05
	After	0.12	25.90	18.37	0.98	0.04	1.14
*Sus scrofa*	Before	0.44	20.82	35.20	4.93	0.51	3.40
	After	0.41	16.73	146.59	2.30	0.60	2.87
*Capricornis milneedwardsii*	Before	0.38	16.52	135.00	3.46	0.22	2.15
	After	0.26	11.18	114.09	1.73	0.45	1.79
*Prionailurus bengalensis*	Before	0.24	17.71	269.45	4.88	0.18	4.48
	After	0.20	16.51	231.71	2.77	0.59	2.42
*Hystrix brachyura*	Before	5.81	88.79	163.41	5.80	0.25	4.49
	After	4.55	36.78	105.39	2.77	0.31	3.27
*Ailurus styani*	Before	0.50	12.60	46.70	3.96	0.07	2.10
	After	0.24	7.40	30.21	1.28	0.14	1.19

*Note:* “Before” refers to the heavy metal content measured in pre‐preservation feces samples, while “After” refers to measurements taken after preservation using the proposed method.

## 4 Discussion

The long‐term preservation of wildlife feces is increasingly important for ecological and conservation research, as fecal samples provide morphological, genetic, and chemical information that supports a wide range of analyses. This creates a growing need for preservation methods that can maintain multiple attributes over longer time scales. In this study, we demonstrate that a multistep preservation method effectively retains the key structural and molecular characteristics of fecal specimens over extended periods, providing a practical solution for long‐term archiving. DNA remained amplifiable and taxonomically informative, and although heavy‐metal concentrations exhibited greater variability across sampling periods, overall elemental profiles were comparable, indicating that trace‐element measurements remain feasible with further methodological refinement. Collectively, these findings demonstrate that long‐term preserved fecal specimens can serve as reliable materials for diverse ecological applications.

Compared with existing preservation approaches, our multistep protocol provides more balanced long‐term protection of morphological, mechanical, and molecular attributes. Conventional methods each have limitations: alcohol fixation preserves DNA well but often alters external appearance and is relatively costly for large‐scale processing (Biswas et al. 2019), while freezing slows degradation but requires strict temperature control and may deform specimens after repeated freeze–thaw cycles. Silica‐gel desiccation is simple but yields brittle specimens prone to mold growth or insect damage over time. Moreover, alcohol immersion can cause partial leaching of water‐soluble or ethanol‐soluble compounds—including certain trace metals—potentially biasing subsequent chemical analyses. In contrast, our protocol avoids prolonged immersion in high‐concentration solvents and instead relies on controlled infiltration, antimicrobial stabilization, and polymer reinforcement, which together produced specimens that remained structurally intact and chemically analyzable after 18 months of storage. This integrated preservation strategy therefore offers improved stability across multiple analytical dimensions and reduces the risk of solvent‐induced artifacts.

The preservation of amplifiable DNA across herbivorous, omnivorous, and carnivorous species indicates broad compatibility with different fecal matrices, which often differ substantially in terms of water content, texture, and inhibitory compounds. Maintaining consistent taxonomic identification over extended storage periods is particularly valuable for ecological studies that rely on archived samples, as it enables retrospective analyses, cross‐year comparisons, and the integration of historical and contemporary datasets. These strengths underscore the potential of preserved fecal specimens to support long‐term monitoring programs and expand the temporal scope of feces‐based molecular research.

Previous studies have demonstrated that feces reliably reflect metal exposure in wildlife, with metals detected from both fresh (Gupta and Bakre 2012; Batista et al. 2024) and frozen samples (Eeva et al. 2020; Queiroz et al. 2025), supporting their broader use in environmental monitoring. Although our method enables the quantification of heavy metals in preserved fecal samples, further refinement and validation are needed to improve measurement consistency across elements and species. Such variability is not unexpected, as heavy‐metal concentrations in feces can be influenced by short‐term dietary changes, sample heterogeneity, and matrix composition, all of which introduce biological noise into chemical analyses and contribute to substantial within‐individual and among‐sample variation (Eeva et al. 2020). In addition, preservation reagents may increase the dry weight of fecal specimens, potentially reducing the apparent concentrations of certain elements. Careful documentation of reagent quantities and concentrations, alongside validation of potential leaching or dilution effects during the infiltration process, will help reduce analytical bias. With continued methodological improvements, the accuracy and stability of heavy‐metal quantification from long‐term preserved fecal specimens are expected to continue improving.

A limitation of our method is the relatively long preparation period; the full six‐step process requires approximately 7 days to complete. However, because the workflow accommodates multiple specimens simultaneously, parallel processing remains feasible even for large sample batches. Although the selected taxa represent herbivorous, omnivorous, and carnivorous mammals, the present dataset does not capture the full diversity of fecal morphologies and digestive physiologies that may influence preservation outcomes. Expanding species coverage will be crucial for evaluating method performance across different dietary types, digestive systems, and ecological contexts. Future work should apply the protocol to broader field surveys and specimen types to increase taxonomic and functional diversity and to assess method generality under varying environmental and biological conditions.

## 5 Perspective: Potential Applications

**FIGURE 4:**
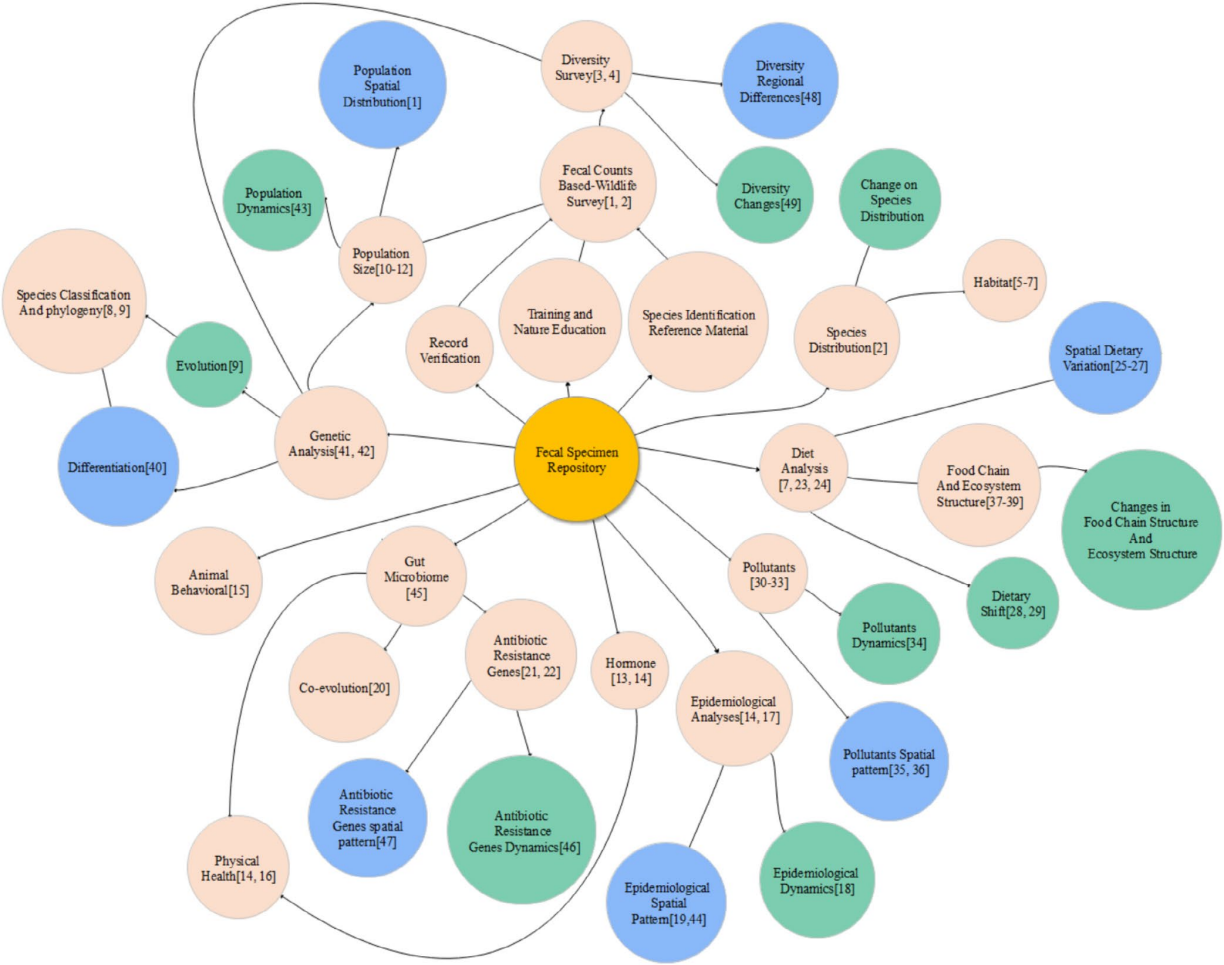
Existing and potential applications of a wildlife fecal specimen repository. Red circles indicate research and application directions, blue circles represent spatial expansion of studies, and green circles denote temporal expansion. The numbers in parentheses correspond to references in fecal research (Appendix S1).

Collecting and preparing biological specimens are fundamental to science, providing irreplaceable records for taxonomy, ecology, and conservation research (Rocha et al. 2014). Building on this principle, the long‐term preservation of fecal specimens transforms an inherently ephemeral material into a durable scientific resource. By retaining morphological, genetic, and chemical integrity over extended periods, curated fecal collections open new avenues for ecological, evolutionary, and conservation research (Figure 4).

Spatially, long‐term preserved fecal specimens enable large‐scale and cross‐regional ecological analyses that are difficult to achieve using fresh samples alone. Stable and verifiable specimens collected from multiple field sites can be integrated into unified datasets, supporting species identification and distribution mapping (Jenkins and Manly 2008; Napolitano et al. 2008), as well as cross‐regional comparisons of habitat use (Bashir et al. 2020; Sand et al. 2021; Seki et al. 2023) and diets (Suter et al. 2004) across geographic regions. Although this study did not directly analyze diet, the successful preservation of DNA indicates that the method is compatible with dietary analyses such as metabarcoding or prey identification, thereby enabling future large‐scale comparative studies of feeding ecology across geographic regions (Shrestha et al. 2022). A standardized fecal specimen repository would therefore greatly strengthen coordinated multi‐site ecological research and improve the assessment of spatial variability in ecological processes, environmental pressures, and conservation outcomes (Knapp et al. 2018; Andersson Stavridis et al. 2024).

Temporally, long‐term preserved fecal specimens enable retrospective and longitudinal ecological analyses that are not possible with fresh samples alone. By maintaining diagnostic morphological traits and genetic material over extended periods, preserved collections support repeated species verification, reduce misidentification, and strengthen the reproducibility of biodiversity assessments through time. This capacity is especially valuable for tracking ecological change, as fecal samples routinely capture information on diet, environmental contaminants, pathogens, and other biological signals that fluctuate in response to ecological conditions. For example, dietary composition exhibits pronounced seasonal and interannual variability (Stenset et al. 2016), while pathogens in feces can reveal the temporal dynamics of disease outbreaks (Gnat et al. 2015; Schilling et al. 2022) and environmental degradation (Malaney and Cook 2018). Consequently, fecal specimen archives provide a foundation for reconstructing past ecological states, detecting long‐term trends in wildlife health and environmental quality, and improving our ability to evaluate ecological responses under accelerating global change.

Beyond spatial and temporal advantages, long‐term preserved fecal specimens provide substantial functional value across diverse research domains. As permanent and verifiable biological materials, fecal specimens offer a reliable foundation for taxonomic reference, species validation, and methodological quality control, thereby enhancing the rigor and reproducibility of ecological studies. These curated collections also serve as practical training tools for field biologists, conservation officers, and wildlife patrol teams, improving in‐field species recognition and reducing misidentification during surveys.

In addition, preserved fecal samples retain a broad suite of biological markers—including structural features, cellular residues, and genetic material—that can support interdisciplinary research. For example, archived specimens allow investigations of gut microbiomes (Menke et al. 2015; Huang et al. 2022), host–microbe interactions (McDonald et al. 2018), and microbial functional traits (Tanca et al. 2017), providing insights into digestive physiology and microbial adaptation. Preserved genetic material also facilitates studies of population structure (Mohanarangan et al. 2022), genetic diversity (Hu et al. 2018), phylogeography (Joshi et al. 2020), and evolutionary relationships (Joshi et al. 2020). Moreover, morphological residues embedded in feces can inform behavioral and ecological research, including foraging strategies (Davis et al. 2015; Murray et al. 2017), habitat use (Li et al. 2024), and species interactions (Lin et al. 2018). Collectively, these functional applications highlight the broader scientific value of establishing standardized fecal specimen repositories, which can substantially expand the scope and analytical capacity of ecological, evolutionary, and environmental research.

## 6 Conclusions

This study presents an effective multistep method for the long‐term preservation of wildlife feces, enabling the stable retention of morphological, mechanical, and genetic attributes across multiple mammalian species. By converting fragile and ephemeral feces into durable and verifiable biological materials, our work addresses a major limitation in ecological research—the lack of standardized, long‐term, cross‐comparable specimens.

Establishing a dedicated fecal specimen repository is therefore not merely an extension of this method, but a foundational step toward building an integrated research infrastructure for ecology and conservation. Such repositories would support rigorous species identification, molecular and chemical analyses, retrospective assessments, and multi‐site ecological monitoring, while also serving as invaluable resources for education, training, and long‐term environmental surveillance. As analytical technologies continue to advance, the scientific value of curated fecal collections will only increase, positioning fecal specimen repositories as essential assets for future biodiversity research and global conservation efforts.

## Author Contributions

**Jiahao Zhang:** conceptualization (equal), data curation (lead), formal analysis (lead), methodology (lead), validation (equal), writing – original draft (lead), writing – review and editing (equal). **Dongling Zhang:** data curation (supporting), formal analysis (supporting), investigation (lead), resources (equal), validation (equal). **Xinrui Xu:** data curation (equal), methodology (equal), project administration (equal), validation (equal). **Yunqiao Zhang:** data curation (equal), methodology (equal), validation (equal), visualization (equal). **Qiang Dai:** conceptualization (equal), funding acquisition (lead), methodology (equal), project administration (equal), resources (lead), supervision (equal), writing – review and editing (equal).

## Funding

This work was supported by the National Natural Science Foundation of China (Grant No. 32470536).

## Conflicts of Interest

The authors declare no conflicts of interest.

## Supporting information


**Appendix S1:** ece372931‐sup‐0001‐Supinfo1.docx.

**Table S1:** BLAST sequence similarity results for fecal DNA extracted at different storage time points from wildlife fecal specimens preserved using the method described in this study, including sequence length, alignment statistics, and corresponding GenBank accession numbers.

## Data Availability

All the required data are uploaded as Supporting Information.
