# Supplementary material for: A Method for Preparing Morphologically Preserved Wildlife Fecal Specimens for Long‐Term Ecological Studies
Source: Ecol Evol. 2026 Jan 11;16(1):e72931. doi: 10.1002/ece3.72931 (PMC12793066; doi:10.1002/ece3.72931)
Supplement: Supplementary file 1 — Appendix S1: ece372931‐sup‐0001‐Supinfo1.docx. [file ECE3-16-e72931-s001.docx]

This appendix lists the key references cited in the study for further reading and verification.

1. Delibes-Mateos, M., et al., Cooke’s index: A simple, cost-effective method for multiple practitioners to estimate European rabbit abundance. Ecological Indicators, 2023. 150: p. 110255.

2. Jenkins, K.J. and B.F.J. Manly, A double-observer method for reducing bias in faecal pellet surveys of forest ungulates. Journal of Applied Ecology, 2008. 45(5): p. 1339-1348.

3. Boyer, S., R.H. Cruickshank, and S.D. Wratten, Faeces of generalist predators as ‘biodiversity capsules’: A new tool for biodiversity assessment in remote and inaccessible habitats. Food Webs, 2015. 3: p. 1-6.

4. Joshi, B.D., et al., From poops to planning: A broad non-invasive genetic survey of large mammals from the Indian Himalayan Region. Science of The Total Environment, 2022. 853: p. 158679.

5. Seki, Y., K. Nakashima, and A. Nakashima, Habitat selection of endangered Amami rabbits on Tokuno-Shima Island in Japan as assessed by counting fecal pellet groups on roads. Animal Biology, 2023. 73(3): p. 259-272.

6. Sand, H., et al., Behavioral effects of wolf presence on moose habitat selection: testing the landscape of fear hypothesis in an anthropogenic landscape. Oecologia, 2021. 197(1): p. 101-116.

7. Bashir, F., et al., Patterns of resource use by Asiatic black bear Ursus thibetanus during pre-hibernation in Kashmir Himalaya, India. Journal of Natural History, 2020. 54(37-38): p. 2455-2469.

8. Werhahn, G., et al., Phylogenetic evidence for the ancient Himalayan wolf: towards a clarification of its taxonomic status based on genetic sampling from western Nepal. Royal Society Open Science, 2017. 4(6): p. 170186.

9. Joshi, B., et al., Revisiting the Woolly wolf (Canis lupus chanco) phylogeny in Himalaya: Addressing taxonomy, spatial extent and distribution of an ancient lineage in Asia. PLOS ONE, 2020. 15(4): p. e0231621.

10. Kohn, M.H., et al., Estimating population size by genotyping faeces. Proceedings of the Royal Society of London. Series B: Biological Sciences, 1999. 266(1420): p. 657-663.

11. Creel, S., et al., Population size estimation in Yellowstone wolves with error-prone noninvasive microsatellite genotypes. Molecular Ecology, 2003. 12(7): p. 2003-2009.

12. WEBBON, C.C., P.J. BAKER, and S. HARRIS, Faecal density counts for monitoring changes in red fox numbers in rural Britain. Journal of Applied Ecology, 2004. 41(4): p. 768-779.

13. Navarro-Castilla, Á., B. Sánchez-González, and I. Barja, Latrine behaviour and faecal corticosterone metabolites as indicators of habitat-related responses of wild rabbits to predation risk. Ecological Indicators, 2019. 97: p. 175-182.

14. Schilling, A.-K., M.V. Mazzamuto, and C. Romeo, A Review of Non-Invasive Sampling in Wildlife Disease and Health Research: What’s New? Animals, 2022. 12(13): p. 1719.

15. Davis, N.E., et al., Power of faecal pellet count and camera trapping indices to monitor mammalian herbivore activity. Wildlife Research, 2022. 49(8): p. 686-697.

16. Mathews, F., et al., Health surveillance in wildlife reintroductions. Biological Conservation, 2006. 131(2): p. 338-347.

17. Gnat, S., et al., Experimental studies of microbial populations and incidence of zoonotic pathogens in the faeces of red deer (Cervus elaphus). Letters in Applied Microbiology, 2015. 61(5): p. 446-452.

18. McDonald, J.L., A. Robertson, and M.J. Silk, Wildlife disease ecology from the individual to the population: Insights from a long-term study of a naturally infected European badger population. Journal of Animal Ecology, 2018. 87(1): p. 101-112.

19. Ulloa, M., Fernández, A., Ariyama, N., Colom-Rivero, A., Rivera, C., Nuñez, P., Sanhueza, P., Johow, M., Araya, H. & Torres, J.C. (2023). Mass mortality event in South American sea lions (Otaria flavescens) correlated to highly pathogenic avian influenza (HPAI) H5N1 outbreak in Chile. Veterinary Quarterly, 43, 1-10.

20. Zhang, Q., et al., Genetic mapping of microbial and host traits reveals production of immunomodulatory lipids by Akkermansia muciniphila in the murine gut. Nature Microbiology, 2023. 8(3): p. 424-440.

21. Hassell, J.M., et al., Clinically relevant antimicrobial resistance at the wildlife&#x2013;livestock&#x2013;human interface in Nairobi: an epidemiological study. The Lancet Planetary Health, 2019. 3(6): p. e259-e269.

22. Greig, J., et al., A Scoping Review of the Role of Wildlife in the Transmission of Bacterial Pathogens and Antimicrobial Resistance to the Food Chain. Zoonoses and Public Health, 2015. 62(4): p. 269-284.

23. Shrestha, B., S. Vařachová, and P. Kindlmann, A Key for Identifying the Prey of Snow Leopard in Nepal Using Features of the Structure of the Hair of Their Prey Present in Their Faeces, in Snow Leopards in Nepal: Predator-Prey System on the Top of the World, P. Kindlmann, Editor. 2022, Springer International Publishing: Cham. p. 75-94.

24. Monterroso, P., et al., Feeding ecological knowledge: the underutilised power of faecal DNA approaches for carnivore diet analysis. Mammal Review, 2019. 49(2): p. 97-112.

25. Murray, M., et al., Greater consumption of protein-poor anthropogenic food by urban relative to rural coyotes increases diet breadth and potential for human–wildlife conflict. Ecography, 2015. 38(12): p. 1235-1242.

26. Havmøller, R.W., et al., DNA metabarcoding reveals that African leopard diet varies between habitats. African Journal of Ecology, 2021. 59(1): p. 37-50.

27. Davis, N.E., et al., Interspecific and Geographic Variation in the Diets of Sympatric Carnivores: Dingoes/Wild Dogs and Red Foxes in South-Eastern Australia. PLOS ONE, 2015. 10(3): p. e0120975.

28. Murray, M.H., et al., Seasonal and individual variation in the use of rail-associated food attractants by grizzly bears (Ursus arctos) in a national park. PLOS ONE, 2017. 12(5): p. e0175658.

29. Stenset, N.E., et al., Seasonal and annual variation in the diet of brown bears Ursus arctos in the boreal forest of southcentral Sweden. Wildlife Biology, 2016. 22(3): p. wlb.00855.

30. Harika, T.L., et al., Fishing Cat Scats as a Biomonitoring Tool for Toxic Heavy Metal Contamination in Aquatic Ecosystems. Toxics, 2023. 11(2): p. 173.

31. Webster, A.B., et al., Optimised ICP-MS quantification method for using animal faeces as a measure of protected area ecosystem health. MethodsX, 2021. 8: p. 101441.

32. Prat-Mairet, Y., et al., Non-invasive monitoring of red fox exposure to rodenticides from scats. Ecological Indicators, 2017. 72: p. 777-783.

33. Wang, S., et al., Feces are Effective Biological Samples for Measuring Pesticides and Flame Retardants in Primates. Environmental Science & Technology, 2020. 54(19): p. 12013-12023.

34. Węgrzyn, M.H., et al., Annual variability of heavy metal content in Svalbard reindeer faeces as a result of dietary preferences. Environmental Science and Pollution Research, 2018. 25(36): p. 36693-36701.

35. Parker, K.H., et al., A heavy burden: Metal exposure across the land-ocean continuum in an adaptable carnivore. Environmental Pollution, 2023. 327: p. 121585.

36. Andersson Stavridis, M., et al., Tracing the footprints of Arctic pollution: Spatial variations in toxic and essential elements in Svalbard reindeer (Rangifer tarandus platyrhynchus) faeces. Science of The Total Environment, 2024. 906: p. 167562.

37. Garfinkel, M., E. Minor, and C.J. Whelan, Using faecal metabarcoding to examine consumption of crop pests and beneficial arthropods in communities of generalist avian insectivores. Ibis, 2022. 164(1): p. 27-43.

38. Mansor, M.S., et al., High-throughput sequencing reveals dietary segregation in Malaysian babblers. Current Zoology, 2021. 68(4): p. 381-389.

39. Coq, S., et al., Faeces traits as unifying predictors of detritivore effects on organic matter turnover. Geoderma, 2022. 422: p. 115940.

40. Calvani, N.E.D., et al., Which species is in the faeces at a time of global livestock movements: single nucleotide polymorphism genotyping assays for the differentiation of Fasciola spp. International Journal for Parasitology, 2020. 50(2): p. 91-101.

41. Ernest, H., et al., Molecular tracking of mountain lions in the Yosemite Valley region in California: genetic analysis using microsatellites and faecal DNA. Molecular ecology, 2000. 9(4): p. 433-441.

42. Reed, J., et al., Molecular scatology: the use of molecular genetic analysis to assign species, sex and individual identity to seal faeces. Molecular ecology, 1997. 6(3): p. 225-234.

43. Prugh, L., et al., Monitoring coyote population dynamics by genotyping faeces. Molecular Ecology, 2005. 14(5): p. 1585-1596.

44. Pleydell, D., et al., Modelling the spatial distribution of Echinococcus multilocularis infection in foxes. Acta tropica, 2004. 91(3): p. 253-265.

45. Videvall, E., et al., Measuring the gut microbiome in birds: comparison of faecal and cloacal sampling. Molecular ecology resources, 2018. 18(3): p. 424-434.

46. Gaire, T.N., et al., Age influences the temporal dynamics of microbiome and antimicrobial resistance genes among fecal bacteria in a cohort of production pigs. Animal microbiome, 2023. 5(1): p. 2.

47. Bengtsson-Palme, J., et al., The human gut microbiome as a transporter of antibiotic resistance genes between continents. Antimicrobial agents and chemotherapy, 2015. 59(10): p. 6551-6560.

48. Prakashbabu, B.C., et al., Eimeria species occurrence varies between geographic regions and poultry production systems and may influence parasite genetic diversity. Veterinary parasitology, 2017. 233: p. 62-72.

49. Massacci, F.R., et al., Inter‐breed diversity and temporal dynamics of the faecal microbiota in healthy horses. Journal of Animal Breeding and Genetics, 2020. 137(1): p. 103-120.
